# Impacts of built environment changes on physical activity in Canada: a systematic review of natural experiments

**DOI:** 10.24095/hpcdp.46.3.04

**Published:** 2026-03

**Authors:** Stephanie A. Prince, Justin J. Lang, Sarah Lawrason, Eric Vallires, Gregory P. Butler, Alison Lake, Gavin R. McCormack

**Affiliations:** 1 Centre for Surveillance and Applied Research, Health Promotion and Chronic Disease Prevention Branch, Public Health Agency of Canada, Ottawa, Ontario, Canada; 2 School of Epidemiology and Public Health, Faculty of Medicine, University of Ottawa, Ottawa, Ontario, Canada; 3 Alliance for Research in Exercise, Nutrition and Activity (ARENA), University of South Australia, Adelaide, South Australia, Australia; 4 Regulatory, Operations and Emergency Management Branch, Ontario Region, Public Health Agency of Canada, Toronto, Ontario, Canada; 5 Regulatory, Operations and Emergency Management Branch, Quebec Region, Public Health Agency of Canada, Montral, Quebec, Canada; 6 Health Library, Public Health Agency of Canada, Ottawa, Ontario, Canada; 7 Department of Community Health Sciences, Cumming School of Medicine, University of Calgary, Calgary, Alberta, Canada

**Keywords:** cycle paths, walkability, transit, parks, schools, pedestrian, active transportation, children, adults

## Abstract

**Introduction:**

The built environment supports physical activity (PA) by providing opportunities to be active in daily life. Natural experiments are valuable for assessing how real-world changes to the built environment affect PA and are critical for guiding policies to improve population-level PA. The objective of this review was to summarize the evidence from natural experiments that investigated the impacts of built environment changes on PA in Canada.

**Methods:**

Searches were conducted in MEDLINE, Embase, PsycINFO, ProQuest Public Health and SportDISCUS, from inception to 27 November 2024. Natural experiment evaluations that included a comparator or historical control group and assessed changes in PA associated with changes in the built environment were eligible. A narrative synthesis summarizes the evidence and the certainty of the evidence.

**Results:**

Results from the included natural experiments (n = 25) suggest positive effects, with low to moderate certainty, of increased walkability, new cycling and pedestrian infrastructure, bike share (bike rental) programs and new trails. However, there was very low to low certainty of no significant effects for bus rapid transit, school building and yard improvements and school zone improvements. Some evidence suggests negative effects of off-leash dog park areas on children’s park-based PA and of daycare yard improvements on moderate-to-vigorous intensity PA.

**Conclusion:**

Few Canadian studies have evaluated the impact of built environment changes on PA, with most emerging in the last decade. Future studies should include larger and more diverse samples and all regions, control for confounders including seasonal variation in outdoor PA, use well-matched control groups and incorporate objective PA measures.

**Highlights**

- Increased neighbourhood walkability, new cycling and pedestrian paths, and bike share programs are the most studied and promising interventions for promoting physical activity.
- New bus rapid transit routes and improvements to school buildings, yards and zones were less studied, and were not as effective at increasing physical activity.
- Few Canadian studies have evaluated the impact of built environment changes on physical activity, and the certainty of the evidence is largely low to very low.
- Natural experiments provide a valuable way to assess the effectiveness of real-world changes to the built environment and are critical for guiding policies to improve population-level physical activity.

## Introduction

Regular physical activity (PA) plays an important role in supporting mental and physical health and well-being.^1^-^4^ The built environment can support PA across the domains of work, school, home, leisure and transportation. The built environment refers to physical environment features that are manufactured or modified by people, including structures and buildings, recreation facilities, green spaces and parks, transportation systems and community design.^5^ The World Health Organization’s *Global Action Plan on Physical Activity 2018–2030* acknowledges the importance of safe and enabling built environments for providing people with opportunities to be physically active in their daily lives.^6^

Systematic reviews have shown that built environment features such as walkability and recreation facilities, parks and green spaces are positively associated with PA;^7^,^8^ this has important implications for health.^5^ However, much of this evidence is cross-sectional, despite the lack of natural experiments being consistently noted as a limitation.^7^-^12^ A recent Delphi study identified the need for stronger study designs, including natural experiments, to establish causality between changes in the built environment and PA.^13^ Natural experiments are interventions that occur in real-world settings where the exposure is not directly manipulated by the researcher.^14^,^15^ Such interventions evaluate the effects of “naturally occurring” changes to the built environment (e.g. bicycle paths, park improvements, light rail transit) on PA. Natural experiments are more practical than traditional experimental studies (such as randomized controlled trials [RCTs]) for investigating the health impacts of environmental interventions where random allocation to exposure is not feasible due to naturally occurring and often large-scale built environment interventions or changes. Natural experiments are critical for advancing the evidence and informing policies to improve population health.^16^,^17^

Previous reviews that focused on natural experiment evidence, regardless of country, have generally found that creating new pedestrian and/or cycling infrastructure (e.g. walking/cycling paths) and public transit including bus rapid transit and light rail transit routes and stops were associated with increased PA including active transportation and total walking and cycling, though not all associations were positive.^10^-^12^,^18^-^21^ Although less studied than pedestrian and cycling path improvements, park and playground improvements (e.g. signage, walking paths, play equipment, seating, waste facilities) have also generally been associated with higher PA or park use.^10^,^12^ Less evidence from natural experiments is available for other built environment changes. 

Using these stronger study designs, compared to cross-sectional studies, results in evidence that is more mixed.^12^ Still, evidence for positive associations between walkability and transport-related PA has been consistent regardless of study design.^7^,^8^ PA has mostly included self-reported measures of general moderate-to-vigorous intensity physical activity (MVPA) or intervention-specific outcomes such as walking and cycling. Most studies have included smaller samples with limited understanding of the representativeness to the general population.

Most evidence has emerged from the United States,^11^,^12^,^18^ and associations between built environments and PA may not always be consistent across countries.^22^,^23^ Country- and region-specific factors such as climate, social norms, culture, geography, topography, socioeconomics, funding and policies influence the built environment and how it is used. PA patterns also differ across countries and global regions.^24^ Canadian sociocultural, geopolitical and behavioural contexts differ from those of other countries, thus country-specific evidence on built environment effects on PA may be more relevant for informing local urban design decisions and policies. Of note, Canadian and American cities are often combined in studies, and while there are similarities in their built environments, such comparisons require careful examination of seemingly comparable parameters.^25^

Several national initiatives have recognized the importance of creating supportive built environments to promote healthy active living in Canada.^26^-^30^ Yet we are aware of only two reviews that summarized Canadian-specific evidence on the associations between built environments and PA.^31^,^32^ Christie et al. found that street connectivity, greenness, destination density and walkability were positively associated with PA among adults living with low socioeconomic status in Canada.^31^ Farkas et al. summarized the literature examining associations between neighbourhood built characteristics and walking among Canadian adults and found that walkability and land use were consistently associated with walking for transportation, while destination proximity was associated with walking for any purpose.^32^

Christie et al. included studies up to 2017^31^ and Farkas et al. up to 2016.^32^ Of these studies, almost all were cross-sectional, thereby precluding the ability to infer causality. Hence, there remains a need for an update to further understand the scope of natural experiment studies of the built environment and PA in Canada. 

Summarizing the effectiveness of built environment changes identifies successful strategies that can improve population PA levels in Canada. In addition, natural experiment evaluations improve our understanding of causality and, accordingly, have gained popularity in the last decade or so.^33^ The objective of this review was to summarize evidence on natural experiments that investigated changes to the built environment and their impact on PA patterns in a Canadian context.

## Methods

The review adheres to the Preferred Reporting Items for Systematic Reviews and Meta-Analyses^34^ and was prospectively registered (http://www.crd.york.ac.uk/PROSPERO/view/CRD42024620614).

***Inclusion criteria***

**Population**

Studies included measurable Canadian data, in urban or rural settings. No age or health restrictions were applied.

**Exposures**

Environmental interventions could include the addition, removal or modification of built environment features or components at macro or micro scales, for example, walking and/or cycling infrastructure, recreation facilities, parks, trails, public transit, traffic-calming features, school design and green spaces. Studies that examined changes in PA as a result of residence relocation were also eligible. Studies examining policies or laws related to the built environment (e.g. vehicle speed limits) were eligible provided these were evaluated in the context of changes in PA. 

Studies examining built environment changes to clinical settings (e.g. hospitals) were not eligible. Only objective changes to the built environment were eligible; self-reported or perceived changes to the environment were not included.

**Controls**

Studies must have included a historical or comparator control. A historical control consists of data collected prior to the change in the built environment, either at the individual or the population level. A comparator control consists of individuals or clusters of individuals not exposed to the same change in the built environment, such as a similar neighbourhood or city without exposure to the intervention.

**Outcomes**

PA included behaviours with an energy expenditure of more than 1.5 metabolic equivalents of task (METs), including time spent in light, moderate or vigorous intensity PA,^35^ as well as step counts. PA could occur across active living domains such as recreation, transportation, occupation or schools and households,^36^,^37^ and could be measured either via self-report (e.g. questionnaire, diary or log, ecological momentary assessment), device (e.g. pedometer, accelerometer, global positioning system [GPS]) or direct observation. PA outcomes could be reported as continuous time (e.g. minutes per day) or counts (e.g. the number of people engaged in PA).

**Study designs**

All natural experiments assessing changes in the built environment were considered, including quasi-experimental, longitudinal interrupted time series, pre–post longitudinal studies, repeated interrupted time series, repeated cross-sectional studies and both prospective and retrospective residential relocation studies. 

**Publication status**

Eligible studies could be peer-reviewed journal articles or indexed dissertations.

**Language**

No language restrictions were imposed on the search strategy, but only publications in English or French were included based on authors’ language knowledge capacity.

**Time frame**

All published literature regardless of date of publication was considered.

***Exclusion criteria***

Studies were ineligible if they were animal studies, focused on a clinical setting (e.g. hospitals), did not report on changes in an environmental feature or an environmental exposure, did not report on changes in a PA outcome (i.e. pre–post or post relative-change measure) or were published in a language other than English or French. Conference abstracts, commentaries, editorials and reviews were also not eligible.

***Search strategy***

A comprehensive search strategy was developed by all the authors and a research librarian [AL]. The primary search was created in MEDLINE (via Ovid), peer reviewed using the Peer Review of Electronic Search Strategies (PRESS) guideline^38^ and translated to Embase (via Ovid), APA PsycINFO and ProQuest Public Health. A second research librarian translated and completed the searches in SPORTDiscus (via EBSCOhost). All searches were run from inception until 27 November 2024. References of topical systematic reviews and included studies were manually searched for additional studies. (See Supplementary Tables 1–5 for the search strategies.)

***Article screening***

Articles were imported into Covidence (Veritas Health Innovation, Melbourne, AU) for screening and duplicates were removed. Two reviewers [SAP, JJL, SL, GRM or GPB], working independently, screened the titles and abstracts to identify potentially relevant articles. The full texts of the potentially eligible studies were then screened by two reviewers [SAP, JJL, SL, GPB or EV], also working independently. If any disagreements arose, they were resolved through discussion, with a third reviewer if necessary.

***Data extraction***

Standardized data extraction forms were piloted and completed in Covidence by two reviewers [SAP, JJL, SL, GPB, EV or GRM], working independently. The reviewers were not blinded to the authors or journals when screening or extracting data, but did not extract data from their own work.

***Risk of bias appraisal***

The risk of bias (RoB) of the individual studies was assessed using the Risk of Bias in Non-randomized Studies of Exposure (ROBINS-E) tool.^39^ Studies were assessed for the following potential biases: confounding; participant selection; exposure assessment; postexposure interventions; missing data; outcome measurement; and selective outcome reporting. For each study, the RoB was reported as low, moderate, serious or critical. RoB assessments were carried out by two reviewers [SAP, JJL, SL, GPB, EV or GRM], working independently, and disagreements were resolved through discussion with a third reviewer [SAP].

***Data synthesis***

A narrative synthesis was used to report findings grouped by built environment change and PA outcome. A meta-analysis was not possible as we did not identify at least two studies reporting on the same built environment change and using the same outcome.

***Grading the overall evidence***

The certainty and strength of the evidence was rated using a modified Grading of Recommendations, Assessment, Development and Evaluation (GRADE) approach.^40^ GRADE provides a transparent and structured process for summarizing the quality of evidence as high, moderate, low or very low. Usually RCTs begin as high quality and other study designs as low; given that RCTs are generally not feasible when evaluating changes to the built environment, for the purposes of this review, natural experiments (i.e. nonrandomized studies) began as high quality.^41^ Quality was based on confidence in the effect estimate and was reduced due to limitations in study design or execution, inconsistency of results, indirectness of evidence, imprecision and publication bias (see Supplementary Table 6 for a summary of decision rules). One reviewer [SAP] assessed the evidence for each built environment intervention and outcome, and the review team verified the assessment for accuracy.

## Results

***Study characteristics***

**Figure 1:**
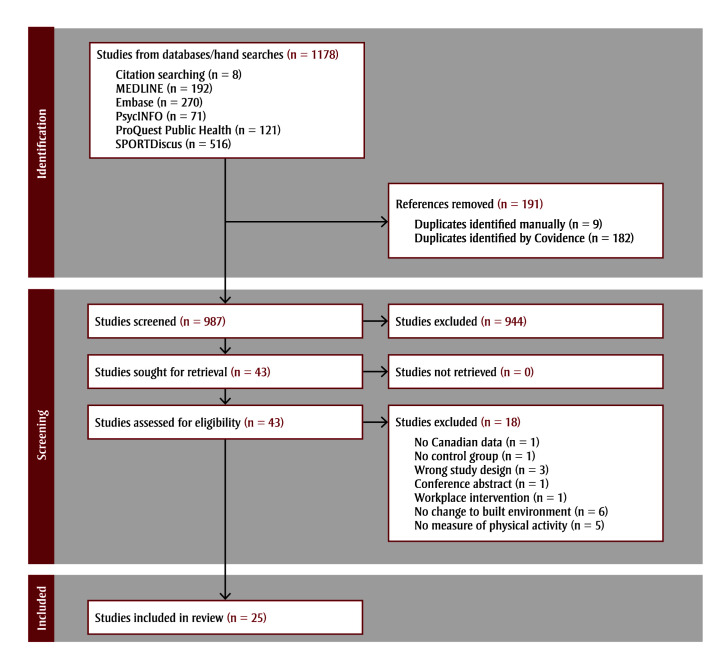
PRISMA flow diagram

**Abbreviation:** PRISMA, Preferred Reporting Items for Systematic Reviews and Meta-Analyses. 

The search identified 1170 potentially relevant papers—192 were identified in MEDLINE, 270 in Embase, 71 in PsycINFO, 121 in ProQuest Public Health and 516 in SPORTDiscus (in order of merging the databases into Covidence). Eight additional papers were identified by manually searching the reference lists in relevant reviews. After removing duplicates, 987 articles were retained for title and abstract screening. Of these, 43 full texts were screened (see Figure 1). 

A total of 25 papers met the inclusion criteria.^42^-^66^ For a list of the 18 excluded full texts and the reasons for their exclusion, see Supplementary Table 7. Characteristics of the included studies are shown in Table 1.

**Table 1:** Characteristics of included studies (n = 25)

First author, year / City or province / Setting	Population description (cohort)	Mean age (SD) or range / Sample size analyzed	Study design / Follow-up time	Built environment change / Comparator	PA outcome (method of assessment measurement)
Change in walkability (via residential relocation)					
Adhikari, 2020^42^ Vancouver, BC Urban	General population: 66.7% F, 67.7% college degree, 20.2% household income < $20 000	35.7 (13.8) years N = 223, I: n = 68, C: n = 155	Residential relocation Average 10 months	I: Increased walkability (residential density, commercial floor area, land-use mix, intersection density) as a result of moving C: Decreased walkability or no change	Total walking trips per day (self-report, travel behaviour survey)
Christie, 2022^45^ Alberta Urban	General population participating in ATP: 64.3% F, 29.0% with low income, 21.2% with high school or lower education	52.5 (9.1) years I1: n = 235 I2: n = 234 C: n = 234 T: n = 703	Residential relocation Mean 2.94 years	Built environment characteristics (population counts, diversity of destinations and street connectivity) and a composite walkability index estimated for 400 m Euclidean buffers around each residential 6-digit postal code at baseline and follow-up; decreased walkability (I1), increased walkability (I2) No change (C)	Total past-week walking time (self-report, IPAQ)
Collins, 2018^47^ Ontario Urban	Imminent movers: 71% F, 80% college diploma or higher	51.2 (15.2) years; High–low: n = 9 Low–high: n = 5 Low–low: n = 19 T: n = 35	Residential relocation Approximately 1 year	Change in neighbourhood walkability measured by Walk Score: low walkability vs. high walkability Pre-move values	Total transportation walking, occupational and recreational PA (self-report, diary)
McCormack, 2017^56^ Calgary, AB Urban	General population: 61.3%–74.5% F, 75.0%–89.5% White, 68.1%–72.2% university educated, 10.2%–19.1% household income < $60 000	I1: 41.6 (16.7) years, n = 47 I2: 42.8 (15.6) years, n = 48 C: 54.4 (13.8) years, n = 820	Residential relocation 12 months	Change in neighbourhood walkability measured by Walk Score at the level of the postal code: decline in walkability (I1); improved walkability (I2) Non-movers vs. maintainers (C)	Perceived direction and relative magnitude of change in total transportation walking, transportation cycling and overall PA (self-report)
McCormack, 2021^58^ Alberta Urban	General population participating in ATP: 61.7%–66.7% F, 58.2%–58.5% completed postsecondary education, 17.1%–24.2% household income ≤ $49 999	I1: 51.8 (8.7) years, n = 165 I2: 51.8 (8.7) years, n = 130 C: 55.7 (9.1) years, n = 5646	Residential relocation 1.5–1.8 years	Moved to neighbourhood with less street integration (I1), moved to neighbourhood with greater street integration (I2); street integration was street connectivity reflecting changes in direction needed to travel between locations in a 1.6 km radial buffer C: Non-movers	Total daily MVPA, active transportation time, leisure VPA, leisure MPA, LW, leisure MPA including walking (MPA+LW), leisure MVPA including leisure walking (MVPA+LW), transportation walking, leisure and transportation walking (self-report, IPAQ)
McCormack, 2023^59^ Alberta Urban	General population participating in ATP: 61.6%–67.2% F, 54.5%–61.6% completed postsecondary education, 17.2%–25.6% household income ≤ $49 999	I1: 51.6 (8.2) years, n = 164 I2: 52.2 (9.6) years, n = 134 C: 55.7 (9.1) years, n = 5679	Residential relocation Median 2 years	Change in neighbourhood walkability index (intersections, destinations, population density) ≤ 400 m buffers around homes: moved to neighbourhoods with less walkability (I1), moved to neighbourhoods with more walkability (I2) Non-movers (C)	Total weekly walking time (self-report IPAQ)
Salvo, 2018^61^ Calgary, AB Urban	General population (Pathways to Health Project): I1: 73.5% F, 69.4% university educated; I2: 49.3% F, 70.8% university educated	I1: 41.6 (16.7) years, n = 49 I2: 42.8 (15.6) years, n = 48 C: NR	Residential relocation Previous 12 months	Change in neighbourhood walkability measured by Walk Score at the postal code level: less walkable (I1), more walkable (I2) Same walkability (C)	Total perceived change in walking time, cycling time, transportation walking, transportation cycling and overall PA (self-reported change)
Wasfi, 2016^64^ All provinces Urban and rural	Urban dwellers (National Population Health Study): 54.4% F, 30.2% completed postsecondary education	38 (9), 18–55 years Movers: n = 1313; non-movers: n = 1663	Residential relocation Every 2 years for 12 years	Cumulative exposure to walkability (Walk Score quartiles) and change in Walk Score quartile between survey cycles (2 years). Dummy variable identified participants who moved 2 or more Walk Score quartiles in either direction (i.e. increase or decrease in walkability) Comparator groups specific to each analysis: low-walkability exposure (quartile 1); movers within same Walk Score quartile	Percent utilitarian walkers (self-report, questionnaire)
New cycling paths and improvements					
Boss, 2018^43^ Ottawa–Gatineau, ON and QC Urban	Strava mobile app users	NR N = 52 123	Repeated cross-sectional 1 year	3 new bike and pedestrian bridge installations: Adwe Crossing, a bike and pedestrian bridge (opened December 2015); Hickory bike and pedestrian bridge (opened August 2015); MacDonald-Cartier pathway (opened December 2015) No comparator or control group. Data collection relied on statistical methods, such as spatial autocorrelation, to distinguish significant changes in ridership patterns from random pattern changes	Cyclist counts on intervention routes (Strava mobile app)
Ling, 2020^53^ Toronto, ON Urban	General cycling population	NA N = 10 579	Longitudinal pre–post Preimplementation cycling counts were collected on average 14 months (range: 9–29 months) before cycle track implementation; postimplementation cycling counts were collected on average 34 months (range: 9–51 months) after cycle track implementation	6 new cycle tracks spanning a total of 8.81 km: Sherbourne St. (2.54 km); Adelaide St. W (1.61 km); Richmond St. W (1.39 km); Simcoe St. (0.69 km); Wellesley St. E (1.28 km) and Wellesley St. W–Queen’s Park (1.30 km) Of these 6 cycle tracks, 3 were upgraded from painted bike lanes: Wellesley St. E, Wellesley St. W–Queen’s Park and Sherbourne St. Only 2 of the 6 cycle tracks were one-way: Richmond St. W and Adelaide St. W. The tracks were separated with mixed uses of bollards, planters, raised curbs and raised tracks Preintervention values	Cyclist counts on intervention routes (direct observation)
Slaney, 2021^62^ Victoria, BC Urban	Local residents: 51.6% F, 73.5% White, 74.4% household income < $50 000	45.7 (13.7) years Baseline: n = 129 Follow-up: n = 153	Longitudinal pre–post 1.5–2 years	Prior to Wave 1 of the study, only the Pandora Ave. protected cycling path in the AAA cycling network was completed; between Waves 1 and 2, 2 protected bike lanes (Fort St. and Wharf St.) and bridge bike lanes (Johnson St. bridge) were added Preintervention values	Daily MVPA (device, SenseDoc [GPS and accelerometer])
Van Veghel, 2024^63^ Hamilton, ON Urban	General population	NR	Longitudinal pre–post 3 years	10 separated cycling infrastructure improvements (painted, plastic and concrete buffers) Preintervention values	Bicycle kilometres travelled on routes (bicycle GPS)
Williams, 2023^62^ Victoria, BC, Kelowna, BC, Halifax, NS Urban	General population: 52%–53% F, 82%–92% White, 12%–20% postgraduate degree, 26%–27% household income < $50 000	≥ 18 years Victoria, BC: n = 842 Halifax, NS: n = 764 Kelowna, BC: n = 826	Quasi-experimental nonequivalent group design with repeated cross-sectional surveys 5 years	Change in cycling infrastructure (km) and AAA cycling infrastructure (km) (including protected bike lanes, off-street paths, local street bikeways) City-level control comparison (difference-in-differences analysis): compared changes in cycling activity between Victoria (intervention city) and Kelowna and Halifax (control cities) over time Proximity-based control comparison (triple-difference analysis): “Exposed” (≤ 500 m of AAA infrastructure) or “unexposed” (> 500 m) to assess differences in cycling activity between the cities over time Control cities selected based on similarities in size; urban layout and climate; recommendations of local government partners; and no plans to build AAA cycling infrastructure networks	Any cycling activity in previous 12 months (yes/no) (self-reports, survey questions)
Multiuse paths					
Frank, 2019^48^ Vancouver, BC Urban	Local residents: 55%–59% F, 77%–86% White, 72%–78% postsecondary education	I: 46.2 years; n = 239 C: 44.7 years; n = 285 T: N = 524	Nonrandomized experimental 2 years	Development of the Comox-Helmcken Greenway (a 2-km long active transportation corridor) with improvements to cycling infrastructure, traffic-calming features, streetscapes, network integration Living > 300 m from the Greenway	Daily total MVPA (self-report, IPAQ)
Frank, 2021^49^ Vancouver, BC Urban	Local residents: 55%–59% F, 77%–86% White, 72%–78% postsecondary education	Median = 44 years; I: n = 239 C: n = 285 T: N = 524	Nonrandomized experimental 2 years	Development of the Comox-Helmcken Greenway (a 2-km long active transportation corridor) with improvements to cycling infrastructure, traffic-calming features, streetscapes, network integration Living > 300 m from the Greenway	Total bicycle use (at least 1 cycling trip), total number of cycling trips (self-report, 2-day travel diary)
Bike share programs					
Fuller, 2013^50^ Montral, QC Urban	General population: 56.7% F, 60.2% college/university educated or greater	49.4 years N = 1803	Repeated cross-sectional 1–2 years	Exposure to bike-share docking stations associated with a bike share program Preintervention and after the bike-share docking stations were removed for the 2 seasons	Past-week cycling time; total, utilitarian and recreational cycling for at least 10 minutes in the past week (self-report, IPAQ)
Hosford, 2018^51^ Vancouver, BC Urban	General population: 51.8% F, 24.4% household income < $50 000	≥ 18 years Weighted preintervention: n = 939 Follow-up 1: n = 841 Follow-up 2: n = 862	Repeated cross-sectional T1: early phase and T2: postimplementation (15 months)	Bike share program (living ≤ 500 m) Preintervention and living > 500 m from bike share program	Any cycling in the past week (self-report, questionnaire)
Bus rapid transit					
Collins, 2015^46^ Kingston, ON Urban	University employees: 66% F, 35% household income < $90 000	NR N = 656	Longitudinal pre–post 1 year	3 new express transit routes that traverse the most common commuter routes in the city Preintervention values	Total active transportation (self-report, survey questions)
McCormack, 2021^57^ Calgary, AB Urban	Local residents: 70% F, 75%–76% completed university, 39.6%–42.5% household income < $99 999	46.8 (13.3) years I: n = 80 C: n = 116	Nonrandomized experimental 1 year	New bus rapid transit stops added ≤ 800 m of residence Residences > 800 m from the rapid transit stops	Total weekly MVPA, walking time, cycling time (self-report)
Off-leash dog park areas					
McCormack, 2016^55^ Calgary, AB Urban	Park visitors: 35.3%–45.2% F	NR Martindale (I1): n = 184 Taradale (I2): n = 167 C1: n = 230 C2: n = 205	Nonrandomized experimental 1 year	Addition of off-leash park areas Parks without designated off-leash park areas	Park-based activities: walking, jogging/running, cycling, dog-related play (direct observation)
New trails					
McGavock, 2019^60^ Winnipeg, MB Urban	General population: 77% White, 61.6% household income > $50 000	18–65 years N = 218	Longitudinal pre–post About 5 months from pre to post	Grooming of a natural frozen waterway as a trail A control time period of 20–30 days prior to and immediately after the intervention was selected to match, as closely as possible, the weather conditions during the intervention	MVPA and steps during trail visit, users of the trail as counts (direct observation and device [PiezoRD])
School building and yard improvements					
Hunter, 2016^52^ Ontario, Alberta 3.4% rural	Secondary school students (COMPASS study): 53.6% F, 73.7% White	15.1 (0.02) years N = 18 777	Nonrandomized experimental 1 year	21 schools made changes to their physical environments. Quantity changes occurred in 5 schools; condition changes occurred in 10 schools; and both quantity and condition changes occurred in 6 schools. 19 schools reported multiple changes that included combinations of changes to recreational programming, use of public health units, the subjective environment/equipment (as reported in the COMPASS School Policies and Practices Questionnaire) and the physical environment (measured using the COMPASS School Environment Application) Preintervention values; 25 schools that made no PA-related changes were collapsed into one control group and served as the reference group	Total weekly MVPA (self-report, questionnaire)
Wong, 2023^66^ Calgary, AB Urban	Grade 1–4 students	NR I: n = 32 C: n = 13	Nonrandomized experimental 16 months postimplementation	Painted designs on tarmac surfaces at 3 intervention elementary schools. The designs were meant to facilitate children’s PA through playing traditional games/activities as well as unstructured play One control school that did not receive painted designs	Total weekly MVPA and steps, observed frequency and intensity of activity on school playgrounds (device [ActiGraph GT3X], direct observation [SOPLAY])
Daycare yard changes to increase nature-based risky outdoor play					
Brussoni, 2017^44^ Vancouver, BC Urban	Children in 2 daycares: 47% F, 69% White	4.3 (0.6) years N = 45	Longitudinal pre–post 2 weeks	Built environment changes that promoted nature-based risky play. The environmental changes addressed the Seven Cs: character (vegetative and natural materials); connectivity (pathways throughout play zones); clarity (well-defined play zones); context (shade); chance (alleys to explore and change encounters); challenge (opportunities for challenge and risky play); and change (arrangements of plants that resulted in group spaces changing with the seasons) Preintervention values	MVPA minutes per 20-minute observation period (device [ActiGraph GT3X])
School zone improvements for active transportation					
Mammen, 2014^54^ All provinces and territories (except Quebec) 9.4% rural	Public elementary school, 38.8% high socioeconomic status	NR	Repeated cross-sectional 1 year	School Travel Planning strategies: 35% capital improvement plans (signage relating to school zones, cross walks, stop signs; bicycle rack installation; sidewalk implementation/improvements); 33% activities (walk-to-school days; walking school bus schemes; interclass walking competitions); 26% education (parent and child safety education; mapping of best routes to school; School Travel Planning promotional materials); 6% enforce (altered drop-off/pick-up zones; presence of crossing guards; traffic/speed calming) None	Rates of active school transportation (self-report hands-up survey)

**Abbreviations: **AB, Alberta; ATP, Alberta’s Tomorrow Project; BC, British Columbia; C, control group; F, female; GPS, global positioning system; I, intervention group; IPAQ, International Physical Activity Questionnaire; LW, leisure walking; MB, Manitoba; MPA, moderate intensity physical activity; MVPA, moderate-to-vigorous intensity physical activity; NA, not applicable; NR, not reported; NS, Nova Scotia; ON, Ontario; PA, physical activity; QC, Quebec; SOPLAY, System for Observing Play and Leisure Activities; T, total; VPA, vigorous intensity physical activity. 

**Note: **All dollar amounts are in Canadian dollars. 

Studies were published between 2013 and 2024 and mainly included middle-aged adults (e.g. 35–57 years) in the general population. A quasi-experimental study design was the most frequently used, followed by residential relocation and pre–post designs. Follow-up times ranged from 3 months to 5 years, with 1 year the most common. 

One study was at the national level (except Quebec)^54^ and another included all the provinces.^64^ Nine studies took place in Alberta,^45^,^52^,^55^-^59^,^61^,^66^ seven in British Columbia,^42^,^44^,^48^,^49^,^51^,^62^,^65^ six in Ontario,^43^,^46^,^47^,^52^,^53^,^63^ two in Quebec,^43^,^50^ one in Manitoba^60^ and one in Nova Scotia.^65^ Aside from the national studies,^54^,^64^ no studies were conducted in the territories, Saskatchewan, Prince Edward Island, New Brunswick or Newfoundland and Labrador. 

Changes in 10 built environment features were evaluated, with most in the context of residential neighbourhoods. The most studied change was increased walkability as a result of residential relocation (31%). New cycling-specific (15%) and combined multiuse (12%) paths were the next most studied changes. PA outcomes were mainly self-reported (64%), measured using devices (20%) or directly observed (12%).

***Risk of bias***

Overall, three studies (12%) had very high risk of RoB, 14 (56%) had high risk of RoB and eight (32%) had some concerns of RoB (see Table 2). No studies were deemed to have a low RoB. The areas with the most concern included a lack of adjustment for important confounding factors (56%) and issues with missing data (52%).

**Table 2:** RoB summary for included studies

First author, year	RoB due to confounding	RoB due to measurement of exposure	RoB due to selection into study	RoB due to postexposure interventions	RoB due to missing data	RoB due to measurement of outcome	RoB due to selection of the reported results	Overall RoB
Change in walkability (via residential relocation)								
Adhikari, 2020^42^	Low	Low	High	Some concerns	High	Some concerns	Some concerns	High
Christie, 2022^45^	Some concerns	Low	High	High	Some concerns	Some concerns	Low	High
Collins, 2018^47^	Very high	Some concerns	High	Some concerns	Very high	Some concerns	High	Very high
McCormack, 2017^56^	Low	High	High	Some concerns	Some concerns	High	High	High
McCormack, 2021^58^	Low	Low	Some concerns	Some concerns	Some concerns	Low	Some concerns	Some concerns
McCormack, 2023^59^	Low	Some concerns	Some concerns	Some concerns	Some concerns	Some concerns	Low	Some concerns
Salvo, 2018^61^	High	Some concerns	Low	Some concerns	Low	Very high	Some concerns	High
Wasfi, 2016^64^	High	High	Some concerns	Some concerns	Some concerns	Some concerns	Low	Some concerns
New cycling paths and improvements								
Boss, 2018^43^	High	Low	Low	High	High	Low	Some concerns	High
Ling, 2020^53^	High	Some concerns	Some concerns	Some concerns	High	High	Low	High
Slaney, 2021^62^	High	High	Very high	High	High	Some concerns	Low	High
Van Veghel, 2024^63^	High	Some concerns	Some concerns	Some concerns	Some concerns	Low	Some concerns	High
Williams, 2023^65^	High	Some concerns	Some concerns	High	Low	Low	Some concerns	Some concerns
Multiuse paths								
Frank, 2019^48^	Some concerns	Some concerns	Low	Some concerns	High	Some concerns	Low	Some concerns
Frank, 2021^49^	Some concerns	Some concerns	Low	Some concerns	High	Low	Low	Some concerns
Bike share programs								
Fuller, 2013^50^	Low	Low	Low	Some concerns	Low	Low	Low	Some concerns
Hosford, 2018^51^	Low	Some concerns	Some concerns	Some concerns	Some concerns	Low	Low	Some concerns
Bus rapid transit								
Collins, 2015^46^	High	Very high	Very high	High	Very high	Low	Some concerns	Very high
McCormack, 2021^57^	Low	Some concerns	High	Low	High	High	Low	High
Off-leash dog park areas								
McCormack, 2016^57^	High	Some concerns	High	Some concerns	Low	High	Low	High
New trails								
McGavock, 2019^60^	Very high	Some concerns	High	Low	High	Low	Some concerns	High
School building and yard improvements								
Hunter, 2016^52^	Low	High	Some concerns	Some concerns	High	High	Some concerns	High
Wong, 2023^66^	Very high	Very high	High	Very high	High	Low	Low	Very high
Daycare yard changes to increase nature-based risky outdoor play								
Brussoni, 2017^44^	High	Low	Some concerns	Some concerns	Low	Some concerns	Low	High
School zone improvements for active transportation								
Mammen, 2014^54^	Very high	Very high	Some concerns	Very high	Very high	Some concerns	Low	High

**Abbreviation:** RoB, risk of bias. 

***Summary of evidence***

Table 3 shows the summary of findings across each built environment change and PA outcome.

**Table 3:** Summary of findings

Summary of effect	No. of participants (no. of studies)	Certainty (quality) of evidence	Interpretation of findings
Positive built environment changes			
Increased walkability (via residential location)			
Walking time Three studies reported a significant increase in transportation walking.^56^^61^^64^ McCormack et al. reported a significant increase in leisure walking.^58^ Adhikari et al. reported a nonstatistically significant ( *p* < 0.1) increase in transportation walking (adjusted for residential self-selection and life events).^42^ McCormack et al. reported no statistically significant change in leisure walking or transportation walking.^59^ Christie et al. reported no significant change in total walking (adjusted for change in marital status and presence of children in the home).^45^ Collins et al. reported a significant decline in transportation walking.^47^	More walkable: n = 667 (+1313 movers unknown) Non-movers: n = 12 719 (8 studies)	Very low certainty RoB: −1 pt, 1 very high, 5 high and 2 some concerns Inconsistency: −0.5 pt, 5 out of 8 studies reported positive effects Indirectness: −1 pt, general populations, but only 1 study adjusted for residential self-selection Imprecision: −0.5 pt, OIS met, but CIs were wide and overlapped, indicating no effect	There is very low certainty of positive effects of moving to a more walkable neighbourhood on walking, especially transportation-related walking.
Recreational PA McCormack et al. reported an increase in leisure MVPA and MPA, but no change in VPA.^58^ Collins et al. reported an increase in recreational PA after moving from a low to a high walkability neighbourhood.^47^	More walkable: n = 135 Non-movers: n = 5646+ (2 studies)	Low certainty RoB: −1 pt, 1 very high, 1 some concerns Inconsistency: 0 pt, both reported positive effects Indirectness: 0 pt, general populations Imprecision: −1 pt, OIS for movers not met	There is low certainty of positive effects of moving to a more walkable neighbourhood on recreational PA.
Total PA Salvo et al. reported no perceived change in total PA.^61^	More walkable: n = 48 Non-movers: NA (1 study)	Low certainty RoB: −1 pt, high Inconsistency: 0 pt, single study Indirectness: 0 pt, general population Imprecision: −1 pt, OIS not met	There is low certainty of no effect of moving to a more walkable neighbourhood on total PA.
Transportation cycling Salvo et al. reported no perceived change in transportation cycling.^61^	More walkable: n = 48 Non-movers: NA (1 study)	Low certainty RoB: −1 pt, high Inconsistency: 0 pt, single study Indirectness: 0 pt, general population Imprecision: −1 pt, OIS not met	There is low certainty of no effect of moving to a more walkable neighbourhood on transportation cycling.
Occupational PA Collins et al. reported no significant change in occupational PA.^47^	More walkable: n = 5 Non-movers: n = 19 (1 study)	Low certainty RoB: −1 pt, high Inconsistency: 0 pt, single study Indirectness: 0 pt, general populations Imprecision: −1 pt, OIS not met	There is low certainty of no effect of moving to a more walkable neighbourhood on occupational PA.
New cycling paths and cycling infrastructure improvements			
Cycling Ling et al. found that the addition of cycling tracks in Toronto, ON resulted in an overall crude increase of 257% in cyclist volume on all affected street segments.^53^ Van Veghel and Scott reported mixed effects of separated cycling infrastructure improvements on bicycle kilometres travelled, with a significant increase in 5 out of 10 intervention routes, a significant decrease in 1 and no change in 4.^63^ Williams et al. examined the impact of an AAA cycling network in Victoria, BC.^65^ Relative to 2 comparator cities (which also saw increases in cycling infrastructure), there was no significant effect on cycling activity. No significant difference in those living closer (< 500 m) vs. further (> 500 m) to the AAA cycling infrastructure was observed in any of the cities.^65^	I: n = 842 C: n = 1590 2 studies reported on cycling counts (3 studies)	Very low certainty RoB: −1 pt, all 3 studies high Inconsistency: −0.5 pt, variation in the direction of effect across studies and sites Indirectness: −1 pt, 2 general populations of local residents and 1 cycling population from a bike share program. Controls were not always free of contamination; the control cities in 1 study increased their cycling infrastructure, and the COVID-19 pandemic likely affected cycling behaviour in all cities Imprecision: 0 pt, OIS met	There is very low certainty of mixed effects of creating new cycling paths and improving existing cycling infrastructure on cycling, with some studies showing increases and others no significant change. Changes may have been affected by location and pre-existing use of infrastructure.
MVPA Slaney found a nonstatistically significant increase in MVPA of 0.93 min/week (95% CI: −5.28 to 7.14) after AAA cycling paths were added.^62^	N = 153 (1 study)	Very low certainty RoB: −1 pt, high Inconsistency: 0 pt, single study Indirectness: −0.5 pt, local population, study suggests high probability of confounding by weather Imprecision: −1 pt, OIS not met	There is very low certainty of no effect of creating new cycling paths on MVPA.
New cycling and pedestrian infrastructure (multiuse pathways)			
Cycling Boss et al. suggested that cyclists shifted to new pedestrian and cycling infrastructure from unprotected routes.^43^ Frank et al. observed a significant greenway–time interaction (IRR = 3.52; 95% CI: 1.54–8.03) and a 252% increase in the rate of cycling trips among residents who resided ≤ 300 m from a new greenway vs. those living > 300 m from the greenway.^49^	Study 1: N = 52 123 users Study 2: N = 524 (I: n = 239) (2 studies)	Low certainty RoB: −1 pt, 1 high, 1 some concerns Inconsistency: 0 pt, both studies suggested increases Indirectness: −0.5 pt, 1 study included Strava app users, the other, local residents’ self-reports Imprecision: 0 pt, OIS met	There is low certainty of positive effect of creating new pedestrian and cycling infrastructure on cycling activity.
MVPA Frank et al. found that retrofitting an urban greenway in a dense downtown neighbourhood resulted in an increase in MVPA among those living ≤ 300 m from the greenway. Living ≤ 300 m from the greenway doubled the odds of meeting the 20+ min/day MVPA recommendation (OR = 2.00; 95% CI = 1.00–3.98).^48^	I: n = 239 C: n = 285 (1 study)	Moderate certainty RoB: 0 pt, some concerns Inconsistency: 0 pt, single study Indirectness: 0 pt, local residents Imprecision: −1 pt, OIS not met	There is moderate certainty of a positive effect of creating new pedestrian and cycling infrastructure on MVPA.
Bike share programs			
Cycling Fuller et al. found that living ≤ 500 m from bike-share docking stations was significantly associated with an increased likelihood of total and utilitarian cycling (OR = 2.86; 95% CI: 1.85– 4.42).^50^ Hosford et al. found that exposure to a bike share program did not result in a change in cycling behaviour among those who only worked or only lived within the service area (≤ 500 m).^51^ Both living and working within the service area was associated with greater odds of cycling at T1 (OR = 2.26; 95% CI: 1.07–4.80) and to a lesser extent, at T2 (OR = 1.37; 95% CI: 0.67–2.83).^51^	N = 2665 (final follow-up) (2 studies)	Moderate certainty RoB: 0 pt, both some concerns Inconsistency: −0.5 pt, both studies showed increases, but there did seem to be differing effect with prolonged exposure Indirectness: 0 pt, local residents Imprecision: 0 pt, OIS met	There is moderate certainty of a positive effect of bike share programs on cycling.
New bus rapid transit routes			
Active transportation Collins and Agarwal examined the effect of adding 3 new bus rapid transit routes and found that while transit ridership among university employees increased by 3%, there was a 0.7% decline in active commuters.^46^ McCormack et al. examined exposure to living ≤ 800 m from new bus rapid transit stops. They found that exposure (over ≤ 12 months) neither influenced time spent in active transportation (walking or cycling) in the neighbourhood nor time spent in regular MVPA.^57^ The authors posit that this may be due to a shift to transit use rather than to active transportation.^57^	Study 1: N = 656 Study 2: I: n = 80, C: n = 116 (2 studies)	Very low certainty RoB: −2 pt, 1 very high, 1 high Inconsistency: 0 pt, similar findings Indirectness: −1 pt, one study was among university employees Imprecision: 0 pt, OIS met	There is very low certainty of no effect of bus rapid transit routes on active transportation. Note that these studies did not account for the potential for multimodal transportation (bus + active travel).
Off-leash dog park areas			
Park PA McCormack et al. explored the effect on visitor activities of adding off-leash areas in parks compared to parks without designated off-leash areas.^57^ They found that addition of off-leash areas potentially lowered the intensity of PA among children, but did not affect adults’ activity. Those visiting parks with dogs participated in less vigorous activity than the visitors without dogs.^57^	I1: n = 184, I2: n = 167, C1: n = 230, C2: n = 205 (1 study)	Very low certainty RoB: −1 pt, high Inconsistency: 0 pt, single study Indirectness: −1 pt, park visitors Imprecision: −1 pt, OIS not met	There is very low certainty of a negative effect of off-leash dog park areas on children’s PA in parks, but no effect on adults’ PA in parks.
New trails			
Trail use, MVPA and steps McGavock et al. explored the effect of grooming a natural frozen waterway to make a trail. During the groomed period there was a 4-fold increase in the number of trail users, who achieved a median of 3852 steps and 23 min/visit of MVPA.^60^	N = 218 (1 study)	Very low certainty RoB: −1 pt, high Inconsistency: 0 pt, single study Indirectness: −1 pt, park visitors Imprecision: −1 pt, OIS not met	There is very low certainty of a positive effect of creating a natural frozen waterway trail on trail use with potential implications for increasing PA.
School building and yard improvements			
MVPA, steps, playground PA Hunter et al. reported on changes to the physical environment of 21 schools. Of the 11 schools that only changed a feature in the built environment (e.g. adding a dance studio, bicycle rack, tennis court, basketball court, fitness/weight room or baseball diamond), average daily MVPA only increased significantly in 1, which had added a bicycle rack.^52^ Wong et al. examined changes in MVPA, steps, and frequency and intensity of school playground activity after designs were painted on tarmac surfaces in elementary schools. Compared to a control school, the 3 intervention schools had significantly fewer minutes of MPA, VPA and MVPA and fewer steps per day. The lowest engagement in PA was found for the designs vs. the playground structures.^66^	Study 1: N = 18 777 Study 2: I: n = 32; C: n = 13 (2 studies)	Low certainty RoB: −2 pt, 1 very high, 1 high Inconsistency: 0 pt, both demonstrate minimal effectiveness Indirectness: 0 pt, students Imprecision: 0 pt, OIS met	There is low certainty of no effect of school built-environment changes (e.g. addition of dance studios, tennis courts, basketball courts, baseball diamonds, fitness/weight rooms, bicycle racks or painted designs on playground tarmac) on student PA.
Daycare yard changes to increase nature-based risky outdoor play			
MVPA Brussoni et al. examined changes in device-measured MVPA during a 20-minute observation period following alterations to a daycare yard to promote nature-based risky play. They found a significant decrease in MVPA 2 weeks after the intervention (−1.32 min; *p * < 0.001), possibly due to an increase in play and social behaviours that did not include MVPA.^44^	45 (1 study)	Very low certainty RoB: −1 pt, high Inconsistency: 0 pt, single study Indirectness: −1 pt, MVPA was determined during one 20-minute observation period Imprecision: −1 pt, OIS not met	There is very low certainty of a negative effect of daycare yard changes to increase risky outdoor play on MVPA.
School zone improvements			
Active transportation to school Mammen et al. reported on School Travel Planning strategies that included improvements to the built environment near and in 35% of 106 schools. No significant change in active school transportation was found at the national level after 1 year, but variation at the school level was considerable. The timing of data collection, i.e. the season, affected rates, which were lower when follow-up occurred during winter.^54^	Sample size not reported (1 study)	Very low certainty RoB: −1 pt, high Inconsistency: 0 pt, single study Indirectness: −1 pt, active school transportation was determined using a hands-up survey Imprecision: −1 pt, OIS unclear	There is very low certainty of no effect of School Travel Planning policies (which included changes to the built environment) on active school transportation.
Negative built environment changes			
Reduced walkability (residential relocation)			
Walking time Three studies reported a significant decrease in transportation walking.^47^^56^^59^ Two studies reported no significant change in transportation walking.^61^^64^ Two studies reported no significant change in leisure walking.^58^^59^ Three studies reported no significant change in total walking.^45^^58^^59^	Less walkable: 669 (+1313 movers unknown) Non-movers: 12 564 (7 studies)	Low certainty RoB: −1 pt, 1 very high, 4 studies high and 2 studies had some concerns Inconsistency: −0.5 pt, variation in direction of effect across outcomes Indirectness: 0 pt, general populations Imprecision: 0 pt, OIS met	There is low certainty of no effect of moving to a less walkable neighbourhood on total or leisure walking. There is low certainty of a negative effect of moving to a less walkable neighbourhood on transportation-related walking.
Recreational PA McCormack et al. reported no change in leisure MPA, VPA or MVPA.^58^ Collins et al. reported no change in recreational PA after moving from a high to a low walkability neighbourhood.^47^	Less walkable: 174 Non-movers: 5646+ (2 studies)	Low certainty RoB: −1 pt, 1 very high, 1 some concerns Inconsistency: 0 pt, 2 out of 2 reported no change Indirectness: 0 pt, general populations Imprecision: −1 pt, OIS for movers not met	There is low certainty of no effect of moving to a less walkable neighbourhood on recreational PA.
Total PA Salvo et al. reported no perceived change in total PA.^61^	Less walkable: 49 Non-movers: NA (1 study)	Low certainty RoB: −1 pt, high Inconsistency: 0 pt, single study Indirectness: 0 pt, general population Imprecision: −1 pt, OIS not met	There is low certainty of no effect of moving to a less walkable neighbourhood on total PA.
Transportation cycling Salvo et al. reported a significant decrease in transportation cycling.^61^	Less walkable: n = 49 Non-movers: NA (1 study)	Low certainty RoB: −1 pt, high Inconsistency: 0 pt, single study Indirectness: 0 pt, general population Imprecision: −1 pt, OIS not met	There is low certainty of negative effect of moving to a less walkable neighbourhood on transportation cycling.
Occupational PA Collins et al. reported no significant change in occupational PA.^47^	Less walkable: n = 9 Non-movers: n = 19 (1 study)	Low certainty RoB: −1 pt, high Inconsistency: 0 pt, single study Indirectness: 0 pt, general populations Imprecision: −1 pt, OIS not met	There is low certainty of no effect of moving to a less walkable neighbourhood on occupational PA.

**Abbreviations: **AAA, All Ages and Abilities; C, control group; CI, confidence interval; I, intervention group; MPA, moderate intensity physical activity; MVPA, moderate-to-vigorous intensity physical activity; NA, not applicable; OIS, optimal information size; OR, odds ratio; PA, physical activity; RoB, risk of bias; VPA, vigorous intensity physical activity. 

***Change in walkability (via residential relocation)***

There is very low certainty of evidence for a positive effect of moving to a neighbourhood with higher walkability on walking (especially transportation-related walking) and recreational PA. There is also low certainty of evidence for no effect of relocating to a neighbourhood of higher walkability on total PA, transportation cycling or occupational PA. There is low certainty for no effect of relocating to a neighbourhood of reduced walkability on total or leisure walking, recreational PA, occupational or total PA, but there is a negative effect on transportation-related walking and cycling. 

Neighbourhood walkability was operationalized using four different measures across the studies. Almost all evidence was from urban-dwelling adults in the general population, with follow-ups from 10 months to 3 years. Most of the evidence was from Alberta, with three studies assessing different outcomes using data from the Alberta’s Tomorrow Project.^45^,^58^,^59^ Wasfi et al. used a different approach in a repeated-measures national longitudinal study over a 12-year period.^64^ Their study was the only one to capture rural respondents, and included more respondents with a less than postsecondary education.

***New cycling paths and improvements***

There is very low certainty of mixed effects of creating new cycling paths or improvements to cycle paths (e.g. improved separation from traffic) on cycling and no effect on MVPA. Outcomes differed across all studies making comparison difficult. Ling et al. found that adding cycle tracks resulted in a 257% increase in cycling volume on affected street segments.^53^ Van Veghel and Scott reported a significant increase in bicycle kilometres travelled in five of the 10 intervention routes with improved cycling paths, a significant decline in one and no change in four.^63^

Williams et al. found no significant impact of developing an All Ages and Abilities cycling network, a separated cycling infrastructure project designed to improve cycling accessibility and safety by building protected bike lanes, off-street paths and local street bikeways in Victoria, BC compared to two control cities or among those who lived closer (≤500 m) rather than further (>500 m) to the cycling paths.^65^ Findings were likely affected by the intervention city completing only about 50% of cycling infrastructure improvements by study’s end, while the control cities built more cycling infrastructure than expected; the COVID-19 pandemic also may have affected cycling behaviour.^65^ The final study examined the same AAA network and found a nonstatistically significant increase of 0.93 min/week in MVPA after the addition of the cycling paths.^62^

***Multiuse paths***

There is low certainty of a positive effect of new multiuse paths on cycling activity and moderate certainty of a positive effect on MVPA. Based on input from cyclists’ Strava apps, which track and record PA, Boss et al. suggested that cyclists used new, multiuse routes rather than unprotected routes.^43^ In two studies, Frank et al. found that the addition of a new greenway resulted in a significant increase in MVPA^48^ and cycling,^49^ especially among residents who lived 300 m or less from the active transportation corridor compared to those living more than 300 m from the corridor.

***Bike share programs***

There is moderate certainty of a positive effect of bike share (or rental) programs on cycling behaviour. Fuller et al. found that exposure to public bike-share docking stations (≤500 m from home) was significantly associated with an increased likelihood of total and utilitarian cycling (odds ratio [OR]=2.86; 95% confidence interval [CI]: 1.85–4.42).^50^ Hosford et al. found that a bike share program did not change cycling behaviour among those who either worked or lived 500 m or less from the service area; only those who both lived and worked 500m or less from the service area had a greater odds of cycling (time 1: OR=2.26, 95% CI: 1.07–4.80; time 2: OR=1.37, 95% CI: 0.67–2.83).^51^

***Bus rapid transit***

There is very low certainty of no effect of new bus rapid transit routes on active transportation, suggesting a potential shift to public transit. In a study of university employees, Collins and Agarwal found that the addition of three new bus rapid transit routes resulted in a 3% increase in public transit, but a 0.7% decline in active transportation.^46^ McCormack et al. found no change in time spent in active transportation after 12 months of living 800m or less from a new bus rapid transit stop.^57^ However, neither study accounted for multimodal transportation (e.g. combined walking or cycling and public transportation for a single trip) in the assessment of the outcome.

***Off-leash dog park areas***

There is very low certainty of a negative effect of off-leash dog park areas on children’s PA, but no effect on adults’ PA. An evaluation found that off-leash dog park areas potentially lowered the intensity of the PA of children visiting the parks, but did not affect adult PA.^55^ In addition, those visiting with dogs engaged in less vigorous PA than those without dogs.^55^

***New trails***

There is very low certainty of a positive effect of new trails on use. One study found that the grooming of a natural frozen waterway trail resulted in a four-fold increase in the number of trail users.^60^ McGavock et al. also used accelerometers to quantify steps (3852 steps/visit) and MVPA (23 min/visit) and suggested that the increase in trail use had potential implications for increasing total PA.^60^

***School building and yard improvements***

There is low certainty of no effect of school-related built environment improvements on student PA. Hunter et al. reported on school changes to the physical environment in 21 schools.^52^ Of the 11schools that only changed features in the built environment (e.g. additions of dance studios, bicycle racks, tennis courts, basketball courts, fitness/weight rooms or baseball diamonds), average daily MVPA only increased significantly in one, which had added a bicycle rack.^52^

Wong et al. examined changes in MVPA, steps, and frequency and intensity of school playground activity after designs were painted on the tarmac surfaces in three elementary schools.^66^ The designs were meant to facilitate children’s PA by encouraging them to play traditional games and activities (e.g. hopscotch, left-right-out, bulls-eye toss, four square) and unstructured games (e.g. random circles of different colours and sizes). Compared to a single control school, the intervention schools had significantly fewer MVPA minutes and steps per day; the lowest engagement in PA was with the tarmac designs and the highest with the playground structures. The authors cited potential school-level factors that may have influenced their use, and the painted designs probably required use instructions and play materials (e.g. balls).^66^

***Daycare yard changes to increase nature-based risky outdoor play***

There is very low certainty of a negative effect of daycare yard changes to increase nature-based risky outdoor play on MVPA. A single study found a significant decrease in MVPA during the single 20-minute observation period (−1.32 min; *p*<0.001) 2weeks after changes to a daycare yard.^44^ Brussoni et al. speculated that this decline was due to an increase in play with natural materials, independent play and prosocial behaviours.^44^

***School zone improvements for active transportation***

There is very low certainty of no effect of School Travel Planning policies on active school transportation. One study found that School Travel Planning strategies that included improvements to the built environment (e.g. signage related to school zones, crosswalks, stop signs, bicycle racks, sidewalk implementation or improvements) were observed near and in 35% of 106 schools.^54^ At the national level, Mammen et al. found no significant change in active school transportation after 1 year, although results varied considerably at the school level. There was also evidence that timing of data collection affected rates, which were lower when follow-up occurred in winter.^54^

## Discussion

In this systematic review we examined evidence on 25 natural experiments designs evaluating built environment changes on PA levels and patterns in Canada. Most of the experiments occurred in Alberta, British Columbia and Ontario, and none focused on rural areas. Most of the studies focused on adults aged approximately 35 to 57 years and had about 1 year of follow-up. Common changes included increased walkability as a result of relocation and the addition of pedestrian or cycling infrastructure. PA outcomes were mostly self-reported and linked to specific interventions such as walkability and walking or cycle paths and cycling.

The results suggest positive effects, with low to moderate certainty, of increased walkability, new cycling and pedestrian infrastructure, bike share programs and trails. However, there was very low to low certainty of no significant effects for bus rapid transit, school building and yard improvements, and school zone improvements. Some evidence also suggests negative effects of off-leash dog park areas on children’s park-based PA and of daycare yard improvements on MVPA.

No studies that fitted our inclusion criteria for study designs examined changes to playgrounds, parks or green spaces, new recreation facilities, aesthetics, lighting, rail transit (light rail, Metro or commuter train), other pedestrian and cycling infrastructure (e.g. benches, bicycle parking) or traffic-calming features.

***Comparisons with the literature across built environment interventions***

Similar to the present findings, non-Canadian studies also show that relocating to a neighbourhood with improved walkability features (e.g. mixed land use, street connectivity) often increases walking.^10^,^11^,^67^ Walking was the most consistent outcome,^67^ though studies are subject to self-selection bias as individuals who are more active may choose to live in walkable areas. In addition, life changes (e.g. childbirth, job changes) may influence PA. Of the eight studies we reviewed, only two^42^,^45^ adjusted for life events and residential self-selection, and only Adhikari et al. found a positive association (*p*<0.1).^42^

A review by McCormack et al. found that associations between the built environment and PA are likely independent of residential location choices, but findings were often attenuated when cross-sectional studies adjusted for neighbourhood self-selection.^12^ Along with our findings, this suggests that life events and self-selection may in part explain changes in walking due to increased walkability. While residential relocation studies provide observational evidence on the potential for changes in walkability and its influence on PA, future studies could benefit from examining effects on PA that might occur with neighbourhood improvements (e.g. gentrification or urban redesign).

Previous systematic reviews that focused on natural experiment studies regardless of country have also generally found that creating new pedestrian and/or cycling infrastructure (e.g. new multiuse paths, cycle paths, bike share programs) increases active transportation and total walking and cycling, though the studies have also shown no significant changes associated with these additions.^10^-^12^,^18^,^19^

A recent systematic review and meta-analysis of primarily multiuse paths in eight countries (1 in Canada) found that PA (almost entirely self-reported) increased by 12% among individuals exposed to new paths (standardized mean difference=0.12; 95% CI: 0.04–0.20).^19^ Another review looked at the effectiveness of changes to the physical environment, including new walking, cycling and multiuse paths, for promoting walking and cycling.^68^ Panter et al. found that only 6 of the 30 studies reported significant positive effects.^68^ The most effective interventions targeted accessibility (e.g. new paths) and safety (e.g. segregation from motor vehicles) and higher quality interventions were more likely to report positive effects.^68^ We also found similar positive effects for multiuse pathways and mixed effects for cycling-specific paths, which all improved accessibility and safety. Some mixed findings can be attributed to pre-existing exposure to infrastructure, location of the infrastructure and a difficulty to account for changes in control cities. Only two Canadian studies evaluated multiuse pathways (the same greenway), and four evaluated cycling-specific infrastructure. Given the recent investments by the Government of Canada to promote active transportation through the National Active Transportation Strategy,^29^ we can expect more opportunities to evaluate the effectiveness of infrastructure projects in the future.

Bike share programs have been less evaluated overall.^69^ They have shown promise for increasing cycling in Canada, as reported in a North American study of bike share programs^70^ that found that living near (<500 m) newly implemented bike share docking stations resulted in increases in cycling over 2 years of follow-up. However, no differences were observed in cities with existing bike share programs relative to cities without such programs. There is some evidence that bike share trips replace those previously undertaken by public transit or walking.^71^ Since cycling is generally higher intensity than walking,^72^ bike share programs may contribute to a greater extent to meeting Canadian PA recommendations. Future Canadian studies are warranted given the increased popularity of e-scooters and e-bikes.

Previous reviews found that new bus rapid transit and light rail transit routes/stops increased PA by 1.76 MET hours/week (approximately 30 min/week of walking or light-to-moderate PA).^20^ One review found that installing a new light rail transit led to a 7% to 40% increase in walking and positive if inconsistent associations with MVPA and cycling.^21^ Our contrasting findings likely reflect a shift to using transit rather than active transportation as the main mode of travel, with bus rapid transit replacing existing public transit options rather than opening new services. Studying measures of multimodal travel, total PA and location-based PA to assess gains in walking and cycling as modes of travel supporting public transit (e.g. walking to and from transit stations) and to discern effectiveness based on pre-existing transit service could be beneficial. The geography of specific stops (population and commercial density) and their amenities (secure bike storage, safe access points) should be taken into account. 

In 2024, the Government of Canada launched the Canada Public Transit Fund to increase the use of public transit and active transportation in Canada.^30^ This fund will provide opportunities to evaluate their effectiveness at increasing PA.

Only single studies explored the remaining built environment interventions (school zone improvements, off-leash dog parks, daycare yard improvements), and more evidence is needed to ascertain the direction and strength of effect. No studies identified park or playground improvements (e.g. signage, recreational areas, play equipment, seating, walking paths, waste facilities), despite previous reviews suggesting conflicting results for such interventions.^10^,^11^,^18^ Similarly, while we found no studies evaluating changes to neighbourhood aesthetics or safety including traffic calming, natural experiments in other countries have found limited evidence that these promote PA.^12^ However, evidence from cross-sectional and observational studies suggests largely mixed associations with PA.^7^,^8^,^73^

***Implications for future research***

Previous reviews have suggested that built environment changes show more positive effects over longer follow-up times,^18^ indicating a need for cumulative exposure and shifts in behavioural norms.^74^ However, we did not identify any clear trends based on follow-up duration. Wasfi et al. found that greater cumulative exposure to higher walkability (i.e. more time spent living in a more walkable neighbourhood) was linked to increased utilitarian walking,^64^ while Hosford et al. showed that a bike share program had short-term effects but less of a long-term impact.^51^ Future evaluations should explore longer-term and cumulative effects of built environment changes.

Certainty in the evidence was mostly very low to low, often because of confounding, postexposure interventions, missing data, exposure assessment, small sample sizes and inconsistency in findings. These limitations, common in natural experiments, highlight the need for additional studies to confirm findings.^10^,^11^,^18^,^75^ Because natural experiments are “real-world” experiments that are challenging to design and implement, many variables are out of the control of the researcher. Nonetheless, the reviewed studies highlight the promise of built environment changes.

While most findings appear to align with review evidence informed from mostly high-income countries, more quasi-experimental research is needed in Canadian contexts to further confirm findings and understand any context-specific implications such as climate, culture and geographic differences. Emerging technologies such as smartphone applications, wearable devices, geolocation devices and artificial intelligence for environmental audits and PA observations allow for easier scaling-up to enable larger samples and greater generalizability.^76^ Used alone, and in conjunction with surveys and place-based research, these technologies offer new opportunities to overcome the limitations of previous approaches, to build on strengths and to provide the robust evidence needed to inform policy.^76^ For example, a recent US study used a large smartphone cohort of more than 2 million and found that increased walkability as a result of relocation was linked to more steps taken and greater MVPA.^77^

The INTerventions, Equity, Research, and Action in Cities Team (INTERACT), a pan-Canadian collaboration, aims to evaluate natural experiments in four Canadian cities, focusing on greenways, cycling paths, bus rapid transit and urban development initiatives.^78^ INTERACT plans to address previous methodological limitations using mobile sensing, accelerometers, geographic information systems and other tools that measure PA and mobility patterns more reliably.

***Strengths and limitations***

This review has several strengths including a preregistered protocol, a comprehensive search strategy created and peer-reviewed by research librarians, RoB assessments to assess study quality and the use of a modified GRADE approach^40^ to assess the evidence certainty. Most previous non-Canadian reviews did not assess the potential for bias from studies and none followed GRADE guidelines. These methodologies help to identify methodological concerns in current studies and a need for higher quality evidence for interventions that can be addressed in future research. 

The limitations are largely due to limitations in the evidence identified. While most samples were drawn from the general population, most evaluations emerged from Alberta, British Columbia and Ontario, and the territories and some provinces were unrepresented. No studies explored rural regions or focused on effects among those who are socially marginalized or disadvantaged (e.g. Indigenous Peoples, 2SLGBTQIA+ communities, racial groups, immigrants or refugees, people with disabilities, older adults), limiting generalizability of findings.

There are unique challenges to engaging with the built environment in Canada, including extreme temperatures and precipitation. While most studies adjusted for season or temperature^43^,^44^,^51^,^52^,^56^,^57^,^61^ or ensured that data collection periods were in the same season,^46^-^49^,^53^,^55^,^58^,^60^ many did not. In addition, most current built environment audit tools do not account for weather and seasonal factors.^79^ While most built environment changes would provide benefit year-round, their use may be challenged by weather conditions (e.g. bicycle paths needing snow removal). Future work should consider this important confounder. 

Although prone to recall and social desirability biases, self-reported measures continue to be the most used. Using devices and novel sources of data would increase reach and improve generalizability. 

Many of the nonresidential relocation studies employed a longitudinal pre–post design with participants’ preimplementation values serving as historical controls. Future work would benefit from including control groups such as comparator sites without the environmental intervention or those living further from the built environment change.

## Conclusion

Few Canadian studies have evaluated the impact of built environment changes on PA, with most emerging in the last decade. Lower-certainty evidence suggests that increased walkability (via residential relocation) and new cycling and multiuse paths and trails are linked to higher PA, while moderate certainty evidence shows that bike share programs increase cycling. There is lower certainty regarding no effect from bus rapid transit initiatives, school building and yard improvements and school zone upgrades. Certainty is also lower regarding negative effects of off-leash dog parks and daycare yards that promote risky play. Future studies should include larger and more diverse samples and all regions, control for confounders including season and residential area selection, use well-matched control groups and incorporate objective PA measures.

## Acknowledgements

We would like to thank Dr. Lindsey Sikora from the University of Ottawa for the search translation into SPORTDiscus.

## Funding

None.

## Conflicts of interest

None.

Justin J. Lang is the journal’s Associate Editor-in-Chief and also one of the Associate Scientific Editors, but recused himself from the review process for this article.

Gavin R. McCormack is one of this journal’s Associate Scientific Editors, but recused himself from the review process for this article.

## Authors’ contributions and statement

SAP: Conceptualization, data curation, formal analysis, investigation, methodology, project administration, writing—original draft.

JJL: Data curation, investigation, methodology, writing—review and editing.

SL: Data curation, investigation, methodology, writing—review and editing.

EV: Data curation, investigation, methodology, writing—review and editing.

GPB: Data curation, investigation, methodology, writing—review and editing.

GRM: Data curation, investigation, methodology, writing—review and editing.

AL: Data curation, resources, methodology, writing—review and editing.

The content and views expressed in this article are those of the authors and do not necessarily reflect those of the Government of Canada.
